# Ras and Rac1, Frequently Mutated in Melanomas, Are Activated by Superoxide Anion, Modulate Dnmt1 Level and Are Causally Related to Melanocyte Malignant Transformation

**DOI:** 10.1371/journal.pone.0081937

**Published:** 2013-12-16

**Authors:** Fernanda Molognoni, Fabiana Henriques Machado de Melo, Camila Tainah da Silva, Miriam Galvonas Jasiulionis

**Affiliations:** 1 Departamento de Farmacologia, Universidade Federal de São Paulo, UNIFESP, São Paulo, Brazil; 2 Departamento de Microbiologia, Imunologia e Parasitologia, Universidade Federal de São Paulo, UNIFESP, São Paulo, Brazil; The Moffitt Cancer Center & Research Institute, United States of America

## Abstract

A melanocyte malignant transformation model was developed in our laboratory, in which different melanoma cell lines were obtained after submitting the non-tumorigenic melanocyte lineage melan-a to sequential cycles of anchorage impediment. Our group has already showed that increased superoxide level leads to global DNA hypermemethylation as well increased Dnmt1 expression few hours after melanocyte anchorage blockade. Here, we showed that Ras/Rac1/ERK signaling pathway is activated in melanocytes submitted to anchorage impediment, regulating superoxide levels, global DNA methylation, and Dnmt1 expression. Interestingly, Ras and Rac1 activation is not related to codon mutations, but instead regulated by superoxide. Moreover, the malignant transformation was drastically compromised when melan-a melanocytes were submitted to sequential cycles of anchorage blockage in the presence of a superoxide scavenger. This aberrant signaling pathway associated with a sustained stressful condition, which might be similar to conditions such as UV radiation and inflammation, seems to be an early step in malignant transformation and to contribute to an epigenetic reprogramming and the melanoma development.

## Introduction

Evidence has linked chronic cellular stress with increased risk for many diseases, including cancer [1]. In melanomas, for example, ultraviolet radiation and persistent inflammation lead to increased reactive oxygen species (ROS) generation and are risk factors for melanoma development [2], [3], [4]. ROS are important regulators of cell signaling, modulating biological processes as proliferation, apoptosis and differentiation. However, misbalance between ROS production and cellular antioxidant system can result in oxidative stress, which may cause damage to DNA, protein and cellular components. Additionally, some studies have also shown that altered ROS levels could affect epigenetic mechanisms [5], [6], [7]. Epigenetic mechanisms promote alterations in gene expression without changes in DNA sequence and comprise DNA methylation, histone modifications and variants, and nucleosome remodeling. These mechanisms are essential to normal development and tissue specific gene expression and their disruption could contribute to cancer development [8], [9]. The most studied epigenetic modification in mammals is the DNA methylation at the 5 position of cytosine residue within cytosine-guanine dinucleotides (CpG), resulting in the formation of 5-methylcytosine [10]. Promoter hypermethylation is linked with gene expression inactivation and results from the activity of a family of DNA methyltransferases (DNMTs), specifically DNMT1, 3A and 3B [11]. Besides epigenetic mechanisms have been heavily studied in cancer, until now it is not known which factors initiate abnormal DNA methylation. In this manner, some authors have proposed that repetitive stress conditions could be a factor driving aberrant epigenetic modifications in cancer development [12], [13]. To better understand the relationship among repetitive injury, epigenetic mechanisms and malignant transformation, our group developed an *in vitro* melanocyte malignant transformation model based on the sustained stress condition exposure. In this model, different melanoma cell lines were obtained after submitting a non-tumorigenic melanocyte lineage, melan-a, to sequential cycles of adhesion impediment [14], [15], [16]. This adhesion impediment was characterized as a stressful condition to melan-a cell line since increased amounts of ROS are produced during this process [5], [17]. Moreover, our group has already showed that global DNA methylation and Dnmt1 protein level increase few hours after melan-a anchorage blockade. Interestingly, this increase is related with increased levels of superoxide anion produced during this condition [6]. Then, next step was to understand how oxidative stress would be linked to Dnmt1 regulation. Would it be through an oncogene signaling? Genes required to constrain tumor development are often inactivated by epigenetic marks, such as hypermethylation of their promoters [18]. However, it is not known whether this silencing occurs by random acquisition of epigenetic marks that confer selective advantages for growth or by activation of specific pathways initiated by an oncogene. Evidences suggest, for example, that repression of tumor suppressor genes linked to DNA methylation is regulated by Ras, an important oncogene [19], [20], [21], known as a gene driving melanoma progression [22], [23]. In this way, the aim of the present work was to understand how superoxide anion could change DNA methylation levels and identify the cellular signaling pathways involved. Here we showed that Ras/Rac1/MEK/ERK signaling pathway is regulated by and regulates superoxide anion production during melan-a anchorage blockade. The activation of this particular signaling pathway culminates in increased Dnmt1 expression and global DNA methylation, which in turn may confer selective advantages for cells submitted to a stressful environment.

## Results

### Scavenging Superoxide Anion Decreases Global DNA Methylation and Dnmt1 Expression

**Figure 1:**
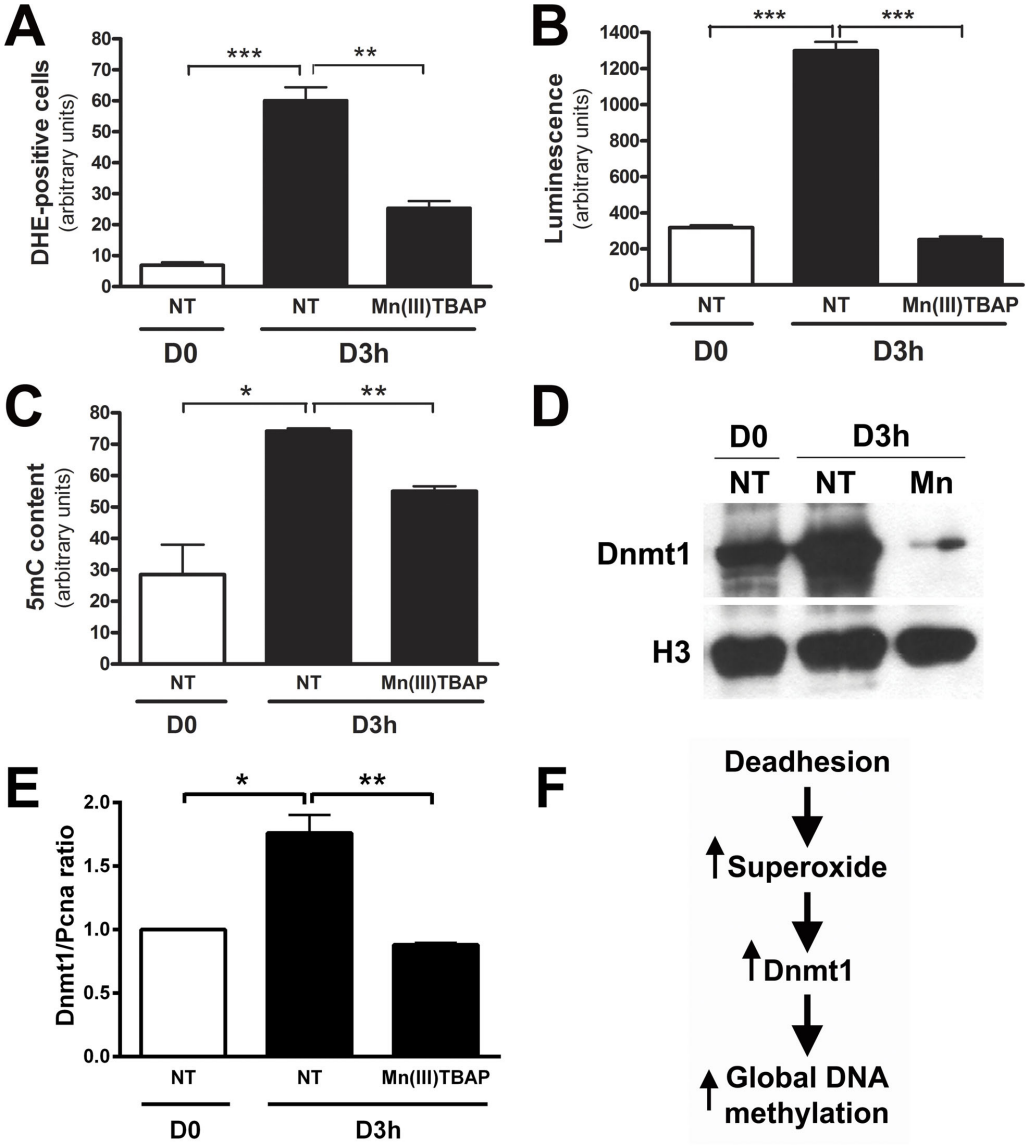
Scavenging superoxide anion abrogates global DNA hypermetylation and increased Dnmt1 levels during melan-a anchorage impediment. Superoxide anion levels was determined by flow cytometry using DHE (**A**) or by luminescence assay using coelenterazine (**B**) in melan-a melanocytes adhered (D0) and submitted to anchorage blockade for 3 h (D3 h), treated or not (NT) with 50 µM superoxide scavenger Mn(III)TBAP. **C.** The total amount of 5-methylcytosine (5 mC) was determined in the same cells by flow cytometry using a specific antibody. **D.** Dnmt1 protein expression was analyzed by Western blot in the same conditions. **E.** Dnmt1 transcript level was determined by real time PCR. **F.** These data show that melanocyte deadhesion leads to superoxide production, which is related to increased Dnmt1 expression and global DNA hypermethylation. For statistical analysis, a non-paired non-parametric Student’s *t*-test was used to analyze differences between the means using GraphPad Prism® version 4 for Windows. The significance level was established at. *p<0.05; **p<0.01; ***p<0.001.

We have previously shown that sequential cycles of melanocyte anchorage impediment result in malignant transformation [16]. This process is a stressful situation for cells and during the first hours of cell-matrix interaction loss, increased superoxide anion and nitric oxide levels, as well alterations in Dnmt1 expression and global DNA methylation were observed [5]. It was also shown that both L-NAME (NOS inhibitor) and N-acetylcysteine (antioxidant) treatment abrogate global DNA methylation increase and Dnmt1 expression observed in melan-a melanocyte lineage submitted to this stressful condition. Surprisingly, L-NAME was shown to decrease superoxide anion but not nitric oxide levels [5], [17]. To elucidate the superoxide anion involvement in the epigenetic alterations found, melan-a cell line was treated with the SOD mimetic Mn(III)TBAP, a superoxide anion scavenger, during anchorage impediment. Scavenging superoxide anion (**Figure 1**, **A** and **B**) results in decrease of global DNA methylation (**Figure 1C**), as well in Dnmt1 protein (**Figure 1D**) and mRNA (**Figure 1E**) level. The reduction of superoxide anion levels in melan-a cells submitted to anchorage blockade in the presence of Mn(III)TBAP was confirmed by both DHE staining (**Figure 1A**) and luminescence using coelenterazine (**Figure 1B**). These data show that superoxide level increases during melanocyte anchorage blockade, leading to increased Dnmt1 expression and global DNA methylation level (**Figure 1F**).

### Activation of Ras Signaling Pathway is Associated with Increase in Dnmt1 Protein Level and Global DNA Methylation

**Figure 2:**
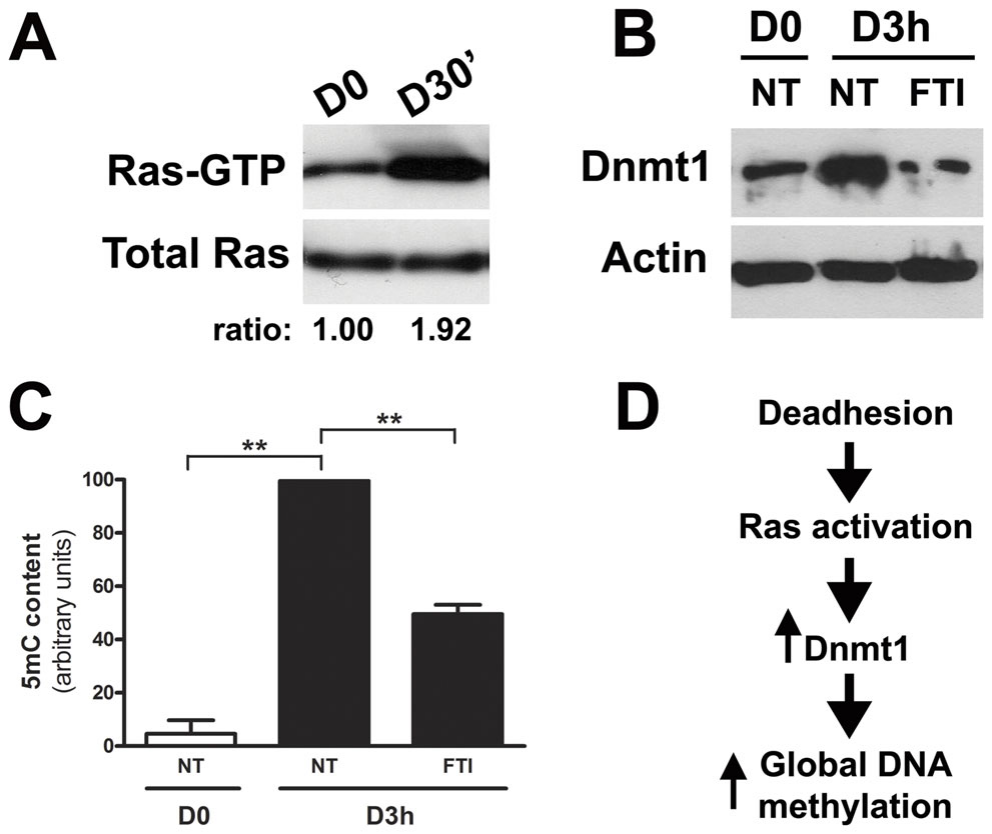
Ras signaling is activated during melanocyte anchorage blockade and its inhibition abrogates increased Dnmt1 protein level and global DNA hypermethylation observed in this condition. **A.** Ras activity was evaluated in adhered (D0) and deadhered melanocytes (D30′) **B.** Dnmt1 protein expression using whole cell extracts was measured in the same conditions. **C.** The 5-methylcytosine (5 mC) content was determined by FACS using a specific antibody in adhered (D0) and deadhered (D3 h) melan-a melanocytes, treated (FTI) or not (NT) with 12.5 µM farnesyltransferase inhibitor. **D.** Melanocyte anchorage impediment results in Ras activation, which leads to increased Dnmt1 level and global DNA hypermethylation. **p<0.01.

Ras signaling pathway is activated in many tumors, including melanomas where it is found frequently mutated [24], and regulates critical events during malignant transformation and progression, as proliferation, apoptosis and drug resistance [25], [26], [27]. As mentioned before, it was demonstrated that modulation of various cellular processes by Ras protein depends on DNA methylation of target genes [20], [28]. Melan-a melanocyte lineage submitted to anchorage blockade shows Ras activation after 30 minutes (**Figure 2A**). However, no codon mutation at 1^st^ and 2^nd^ exons (where mutations frequently occurs) was found in melan-a cell line (data not shown). Treating melan-a cell line submitted to anchorage impediment for 3 hours with FTI (farnesyltransferase inhibitor and Ras inhibitor) abrogates the increase in Dnmt1 protein level (**Figure 2B**) and restricts global DNA hypermethylation (**Figure 2C**), pointing to the involvement of Ras signaling in the alteration of methylation patterns during early events involved in malignant transformation associated with sustained stress (**Figure 2D**).

### Rac1 and Ras, Considered Driver Genes in Melanomas, are Activated during Melanocyte Anchorage Blockade, and Regulate and are Modulated by Superoxide Anion

**Figure 3:**
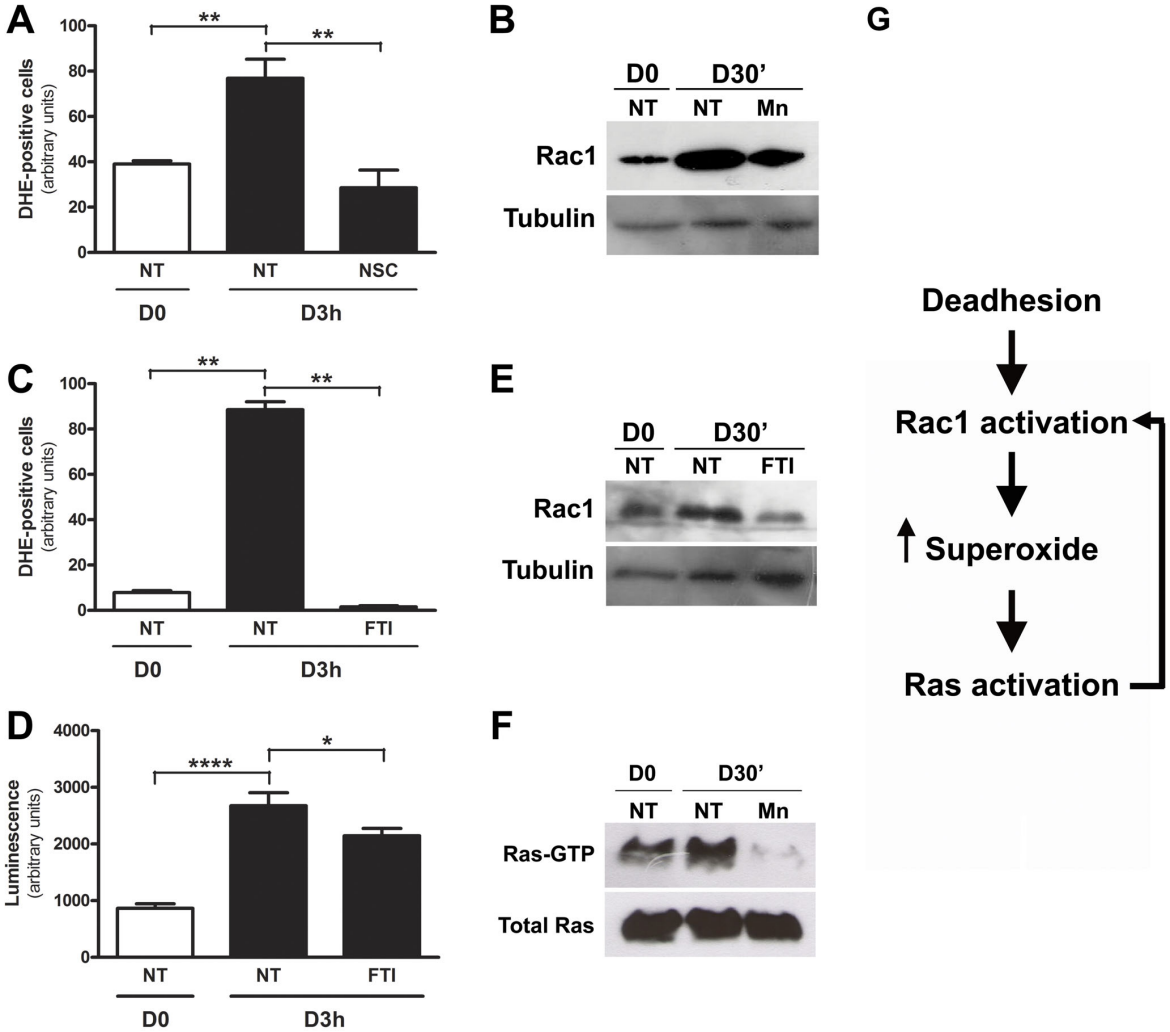
Rac1 and Ras, considered driver genes in melanomas, are activated during melanocyte anchorage blockade, and regulate and are modulated by superoxide anion. **A.** Melanocytes were submitted to anchorage substrate impediment (D3 h) in the presence or not (NT) of 100 µM NSC, a Rac1 inhibitor, and superoxide anion levels were evaluated by flow cytometry using DHE. **B.** Translocation of Rac1 to membrane was evaluated by Western blot using membrane-enriched extracts in adhered (NT) and deadhered melanocytes, treated or not with 50 µM superoxide scavenger Mn(III)TBAP (Mn). Superoxide anion was determined in adhered (D0) and in melan-a cells submitted to anchorage blockade for 3 hours (D3 h) in the presence or not (NT) of 12.5 µM FTI by flow cytometry using DHE (**C**) or by luminescence assay using coelenterazine at same condition (**D**). **E.** Rac1 membrane translocation was evaluated by Western blot in adhered (NT) and deadhered melanocytes, treated or not with 12.5 µM FTI. **F.** Ras activity was evaluated in adhered (D0) and deadhered melanocytes (D3 h) treated (Mn) or not (NT) with 50 µM Mn(III)TBAP. **G.** Rac1 is activated during deadhesion, contributing to superoxide production and Ras activation. Activated Ras modulates Rac1 activation and superoxide anion level in a feedback mechanism. For statistical analysis, a non-paired non-parametric Student’s *t*-test was used to analyze differences between the means using GraphPad Prism® version 4 for Windows. The significance level was established at. *p<0.05; **p<0.01; ***p<0.001.

Previous data from our group identified uncoupled endothelial nitric oxide synthase as one of the superoxide anion sources during anchorage impediment [17]. Here, NADPH oxidase was identified as another source of superoxide anion in melan-a melanocytes submitted to anchorage restriction, since NSC23766 (RAC1 inhibitor) abrogated superoxide levels (**Figure 3A**). Rac1, a small Rho GTPase, is a NADPH oxidase cytosolic component that is recruited to membrane after activation by Ras [29]. Increased Rac1 protein level in membrane-enriched extracts was observed in melan-a melanocytes submitted to adhesion blockade for 30 minutes (**Figure 3B and E**). This increase was inhibited by FTI showing that activation and translocation of Rac1 to membrane is downstream to Ras **(** **Figure 3E** **)**. Moreover, the recruitment of Rac1 to membrane was also inhibited in the presence of superoxide scavenger Mn(III)TBAP, indicating a feedback regulation mechanism **(** **Figure 3B** **)**. Recently, two independent groups demonstrated that Rac1 is the third driver gene more frequently mutated in melanomas after Ras and B-Raf [22], [23]. Nevertheless, there were no point mutations in the whole Rac1 translated sequence (data not shown), indicating that its activation is by another mechanism. Melan-a melanocytes submitted to anchorage blockade in the presence of FTI showed decreased superoxide anion levels (**Figure 3** **, C** and **D**), indicating that activated Ras signaling pathway in melan-a maintained in suspension regulates ROS levels. Increased ROS generation is associated with activation of several oncogenes, including Ras, acting as second messengers and modulating transduction pathways [30], [31]. In order to investigate if superoxide anion regulates Ras activation, melan-a cells were treated with superoxide scavenger Mn(III)TBAP and submitted to anchorage blockade for 30 minutes, when Ras activation occurs. **Figure 3F** shows that superoxide anion depletion inhibited Ras activation, suggesting a feedback regulatory mechanism (**Figure 3F**).

### ERK Activation Downstream to Ras Induces Increased Dnmt1 Protein Expression during Anchorage Blockade

**Figure 4:**
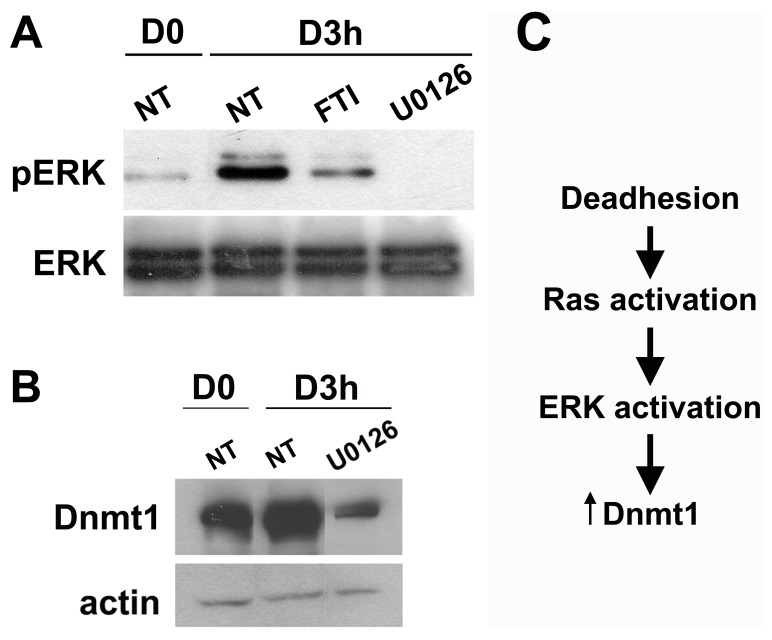
ERK activation downstream to Ras induces increased Dnmt1 protein expression during anchorage blockade. **A.** ERK activation was analyzed by Western blotting in adhered (D0) and deadhered (D3 h) melanocytes treated or not (NT) with 12.5 µM FTI or 15 µM U0126. **B.** Dnmt1 protein expression using whole cell extract was measured in adhered (D0) and deadhered (D3 h) melanocytes treated or not (NT) with 15 µM U0126. **C.** Anchorage impediment leads to Ras/ERK activation, which results in increased Dnmt1 protein level.

The small GTP-binding protein Ras regulates activation of several signaling pathways including Raf/MEK/ERK [32]. ERK activation was observed 30 minutes after melan-a anchorage blockade (data not shown) and maintained until 3 hours in this condition (**Figure 4A**). ERK activation was abrogated using FTI, confirming that ERK activation is downstream to Ras, and was inhibited using U0126, a specific MEK inhibitor (**Figure 4A**). ERK is downstream to Ras signaling pathway and its participation in epigenetic regulation was demonstrated by other authors [33]. Then, we next examined the effect of ERK inhibition on global DNA methylation and Dnmt1 protein level during melanocyte anchorage blockage. Our results showed that treatment with ERK inhibitor abrogated significantly the increase in Dnmt1 protein level observed during melan-a anchorage impediment (**Figure 4B**). PI3K-AKT signaling pathway can also be regulated by Ras activation [34]. This pathway was also activated in melanocyte cells submitted to anchorage blockade (data not shown). However, AKT inhibition was not able to abrogate either the increase in Dnmt1 protein level and superoxide anion level during loss of cell-matrix interactions (**Figure S1**). These data indicate that melanocyte anchorage blockade leads to ERK activation downstream to Ras, which results in the increase of Dnmt1 protein level (**Figure 4C**).

### ERK Signaling Pathway is Regulated by Superoxide Anion in a Feedback Mechanism

**Figure 5:**
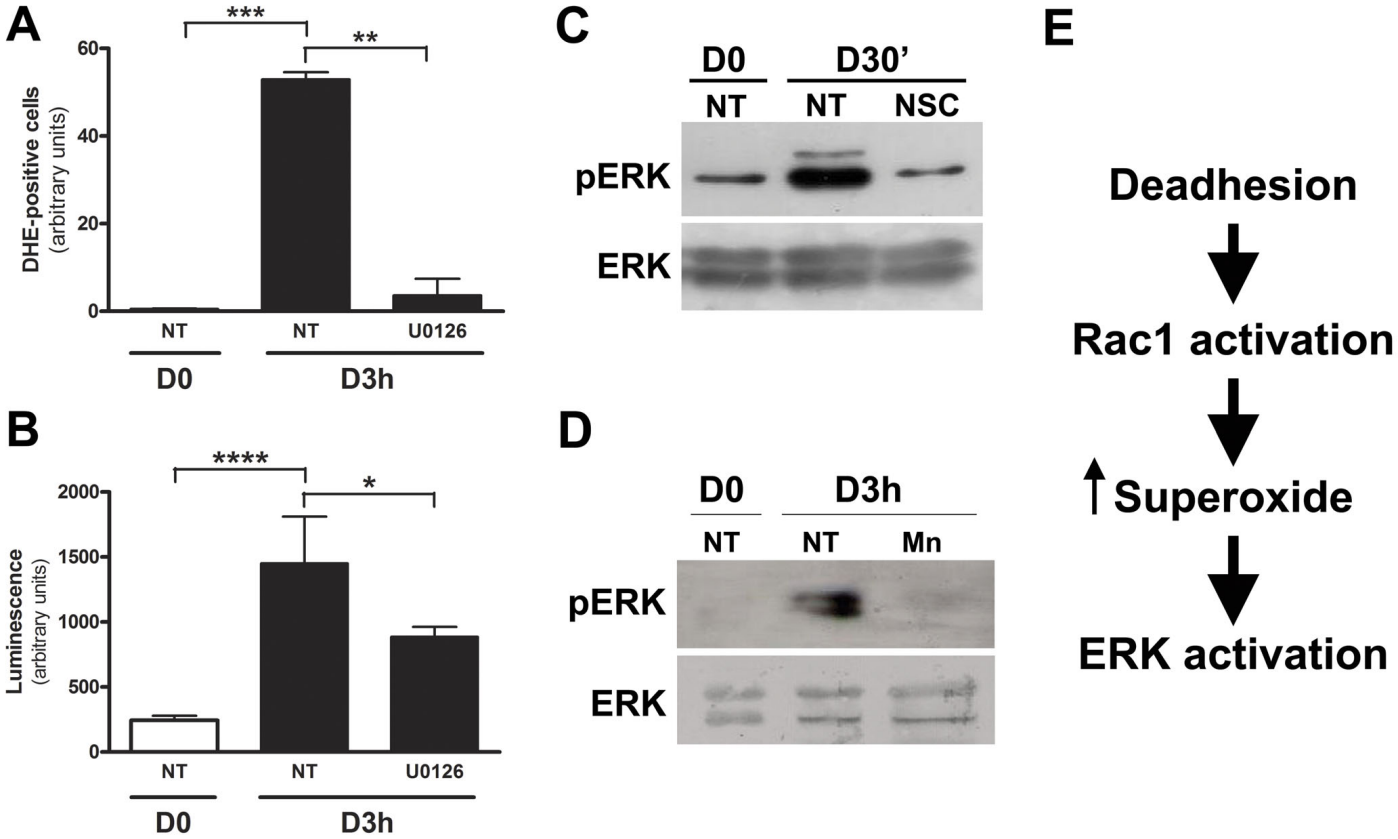
ERK signaling pathway is regulated by superoxide anion in a feedback mechanism. Superoxide anion levels was analyzed in adhered (D0) and deadhered (D3 h) melanocytes in the presence or not of 15 µM U0126 by flow cytometry using DHE (**A**) or by luminescence assay using coelenterazine (**B**). ERK activation was analyzed by Western blotting in adhered (D0) and deadhered (D3 h) melanocytes treated or not (NT) with 100 µM Rac1 inhibitor NSC (**C**) or 50 µM Mn(III)TBAP (**D**) **E.** ERK activation induced by anchorage impediment is downstream of Rac1 activation and superoxide production. *p<0.05; **p<0.01; ***p<0.001; ****p<0.0001.

Several studies have showed the involvement of ERK activation in reactive oxygen species production [35], [36]. Then, to determine if ERK signaling pathway regulates superoxide production during anchorage blockade, melan-a cell line was treated with U0126 during adhesion impediment and superoxide anion levels were evaluated using DHE or coelenterazine. **Figure 5** (**A** and **B**) shows decrease in superoxide generation after inhibition of ERK activation. ERK activation seems to be downstream to Rac1 membrane translocation and superoxide anion production, since NSC, a Rac1 inhibitor, abrogated ERK phosphorylation (**Figure 5C**). Additionally, melan-a cell line treated with superoxide scavenger Mn(III)TBAP during adhesion impediment showed inhibition of ERK phosphorylation (**Figure 5D**), indicating the role of superoxide anion in ERK activation and suggesting a feedback mechanism between ERK and superoxide anion.

### Abrogating Superoxide Anion during Sequential Cycles of Anchorage Restriction Results in Clones with Reduced Cell Proliferation and Clonogenicity and Increases the Time Required to Malignant Conversion

**Figure 6:**
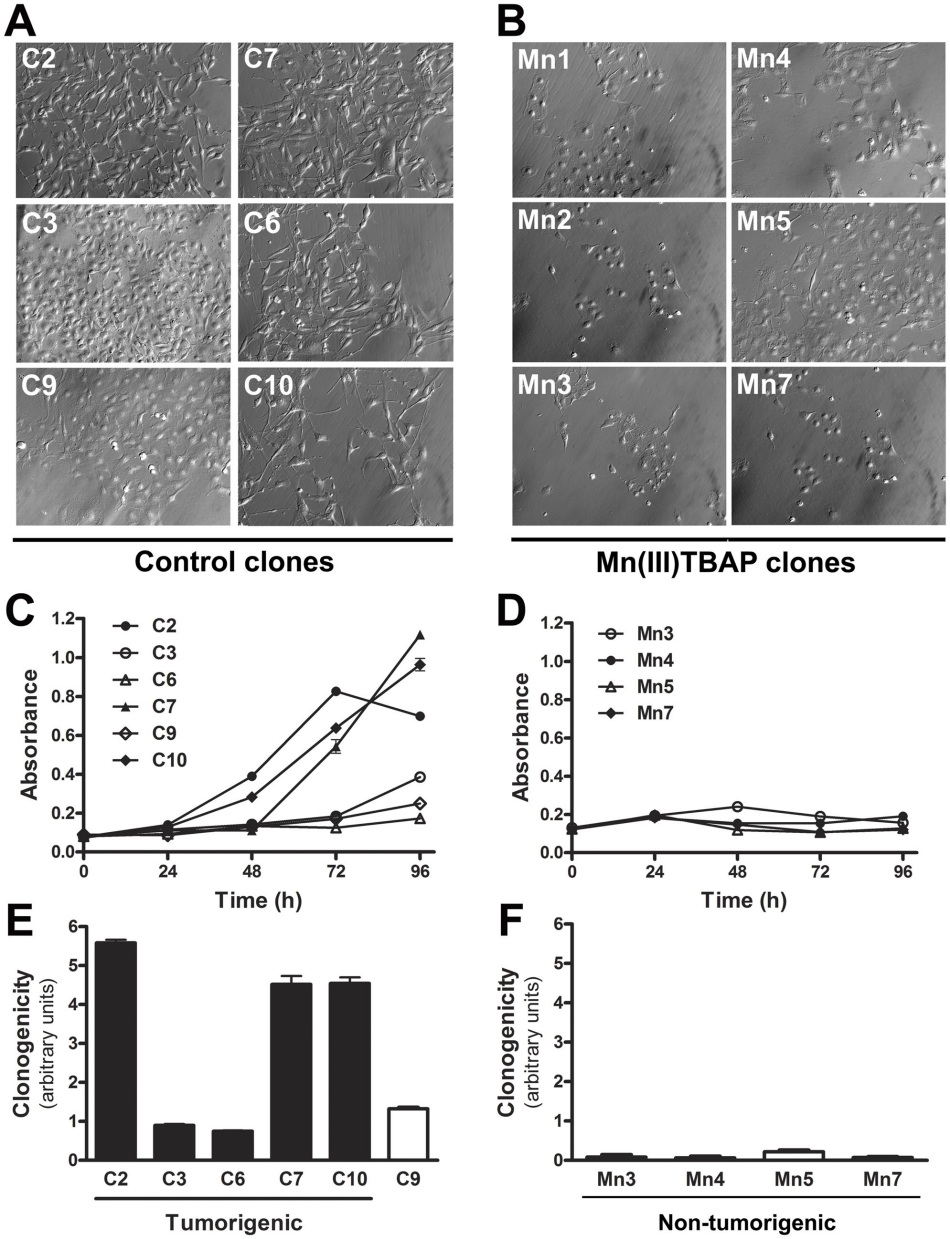
Abrogating superoxide anion during sequential cycles of anchorage restriction results in clones with reduced cell proliferation and clonogenicity and increases the time required to malignant conversion. Melan-a melanocytes were submitted to sequential cycles of anchorage impediment in the presence (Mn clones) or not (control clones) of 50 µM Mn(III)TBAP. Almost control clones showed spindle morphology (**A**) whereas Mn clones senescent-like aspect (**B**). Cell proliferation was analyzed by MTT assay in control (**C**) and Mn clones (**D**). The clonogenic capability of control (**E**) and Mn clones (**F**) was evaluated before escape from senescent-like phenotype by plating 200 cells on 60 mm-dishes. After 9 days, the cell number was estimated by measuring the absorbance after lysing the cells stained with Toluidine blue.

After inappropriate attachment to extracellular matrix, superoxide anion level increases in melan-a melanocyte lineage [5], [17], fibroblasts, endothelial and epithelial cells [37], [38]. Several studies have shown the fundamental role of superoxide anion in cell survival and *anoikis* resistance through the modulation of different signaling pathways [39], [40]. In this way, we performed the sequential cycles of anchorage blockade in presence of superoxide scavenger Mn(III)TBAP. We observed an increased latency time for the establishment of clones in the presence of superoxide scavenger after limiting dilution (**Table 1**). Two weeks after the last cycle of substrate anchorage impediment, virtually all control clones (cycles of anchorage impediment in the absence of Mn(III)TBAP) obtained by limited dilution were viable. Differently, only six clones obtained after anchorage blockade cycles in the presence of Mn(III)TBAP were viable. Clones obtained both in the presence or absence of Mn(III)TBAP were maintained in culture to observe their behavior. Then, after 60 days in culture, all control clones were at fourth passage (p4) and three of them, named C2, C7 and C10, presented altered morphology (spindle shape) **(** **Figure 6A** **)** as that presented by 4C3+, 4C11+, Tm1 and Tm5 metastatic melanoma cell lines established by our laboratory using the same anchorage substrate impediment approach [16]. These clones (C2, C7 and C10) presented high cell proliferation capacity *in vitro*, while other three (C3, C6 and C9) apparently had a senescent-like morphology characterized by increased cell size, flattened and thin cytoplasmic appearance and slow growth in culture conditions (**Figure 6A**). However, C3 and C6 were maintained in culture for more 34 days (totalizing 94 days), until 16^th^ passage (p16), when they lost senescence-like morphology and decreased their doubling time. Cell proliferation assays showed that all control clones, including C9, have higher proliferative rate when compared with Mn(III)TBAP clones **(** **Figure 6** **, C** and **D)**. Moreover, clonogenic assays were performed with control and Mn(III)TBAP clones after 94 days, when control cell clones lost senescent-like morphology. All control cell lines were able to form colonies, while none Mn(III)TBAP lineages which have not escaped from senescence-like phenotype, had this capability (**Figure 6** **, E** and **F**). In order to test their *in vivo* growth capacity, the control clones C2, C7 and C10 (p4), C3 and C6 (p16) were inoculated in syngeneic mice and were tumorigenic with different times for tumor development (**Table 1**). In an opposite way, all Mn(III)TBAP clones presented senescent-like morphology until 135 days maintained in culture **(** **Figure 6B** **)**. Mn2 clone, for example, altered its morphology at 12^th^ passage, only after 135 days in culture and two clones designated Mn1 (p13) and Mn7 (p17) escaped from senescence after 195 days. However, after this period Mn3, Mn4 and Mn5 clones have not lost the senescent-like morphology. Additionally, all Mn(III)TBAP clones were subcutaneously injected into syngeneic mice and only clones Mn2 and Mn7, only after loss their senescent-like morphology, were tumorigenic.

**Table 1:** Characteristics of control and MnTBAP clones.

		Before senescent-like phenotype	After senescent-like phenotype		
Cell lines	Latency time for senescent-like phenotype escape(days)	Latency time for tumor development (days)*	Clonogeniccapability	Latency time for tumor development (days)*	Clonogeniccapability
**Control clones**					
**CL2**	60	13	Yes	13	Yes
**CL3**	94	Non-tumorigenic	ND	60	Yes
**CL6**	94	Non-tumorigenic	ND	18	Yes
**CL7**	60	15	Yes	15	Yes
**CL9**	∞	Non-tumorigenic	ND	Non-tumorigenic	No
**CL10**	60	15	Yes	15	Yes
**MnTBAP clones**					
**Mn1**	135	Non-tumorigenic	No	Non-tumorigenic	Yes
**Mn2**	135	Non-tumorigenic	No	15	Yes
**Mn3**	∞	Non-tumorigenic	No	Non-tumorigenic	No
**Mn4**	∞	Non-tumorigenic	No	Non-tumorigenic	No
**Mn5**	∞	Non-tumorigenic	No	Non-tumorigenic	No
**Mn7**	195	Non-tumorigenic	No	16	Yes

×10^6^ cells subcutaneously inoculated; ND – Not determined; ∞ - Cells that until now have not escaped from senescent-like phenotype. 1

## Discussion

Misbalance between reactive oxygen species production and antioxidants is believed to cause or contribute to several human diseases, such as cancer. Another common deregulated process in tumor cells is DNA methylation, which affects gene expression and chromatin stability. Many tumor suppressor genes became hypermethylated in cancer and a currently explanation is the increased expression of DNMT1 observed in several tumor cell lines [41], [42]. Nevertheless, the factors regulating DNMT1 expression and contributing to the initiation of epigenetic aberrant modifications are still unknown. Growing evidences support the link between pathological ROS production and epigenetic aberrant alterations found in cancer [13], [43]. In this work, Ras/Rac1/ERK pathway was identified as the signaling pathway involved in the process that culminates in increased superoxide anion level, Dnmt1 protein overexpression, increased global DNA methylation level, and melanocyte malignant transformation. Normally, cell response to pathological ROS levels activates numerous intracellular signaling pathways, which in turn regulate transcriptional changes that allow cells to respond appropriately to new environment. Several authors have shown that activation of Ras signaling pathway leads to increased intracellular ROS levels in different cell lines, such as keratinocytes and epithelial cells [44], [45], [46]. Although the above studies implicate Ras signal transduction in the generation and regulation of intracellular superoxide anion levels, there are few evidences that Ras can be also activated by ROS [47], [48], [49]. In NIH-3T3 fibroblasts, the constitutively activated isoform of p21^Ras^, H-Ras^v12^, increased superoxide production by Nox1 (NADPH oxidase catalytic subunit) and was found to be functionally required for cellular transformation [50]. It was also shown that activated Ras induced the translocation of NADPH oxidase cytosolic components (p67^phox^ and Rac) to plasma membrane leading to its activation [51], [52]. NADPH oxidases are a family of five-subunit enzymes that transfers electrons from NADPH to molecular oxygen to produce superoxide and its metabolite, hydrogen peroxide. In fact, we found increased Rac1 translocation to membrane (**Figure 3** **, B** and **E**) and Ras activation (**Figure 2A**) during melan-a anchorage impediment and part of superoxide generated by this process seems to come from NADPH oxidase, since NSC, a Rac1 inhibitor, decreased superoxide levels (**Figure 3A**). Additionally, superoxide production showed to be affected by Ras-ERK signaling during loss of cell-matrix attachment, once FTI and U0126 treatment abrogated its production (**Figures 3C, 3D**, **5A and 5B**). In the other hand, superoxide anion also affects Ras-ERK pathway since its depletion impaired their activation during anchorage restriction (**Figures 3F** and **5C and 5D**). It was shown that oxidants as H_2_O_2_ can direct modify H-Ras Cys118, which resides on a loop close to guanine nucleotide, affecting GTP/GDP exchange and increasing GTP loading [53]. It was demonstrated a landscape of driver coding mutations associated with UV-light-induced damage in human melanoma, which include oncogenes as Ras, B-Raf and Rac1 [22]. Here we show that Ras and Rac1, oncogenes that driver deregulated process such as proliferation and apoptosis in melanoma development, can be activated by reactive oxygen species without any known mutation. Oxidative stress-induced ERK1/2 activation is reported in different cell types [54], [55]. Activation of ERK by ROS plays a critical role in apoptosis protection, since antioxidants decrease ERK phosphorylation and induce tumor cell death [37].

Besides modulation of signal transduction, redox-dependent transcriptional regulation occurs through reversible oxidation of numerous proteins, including tyrosine phosphatases, tyrosine kinases and transcription factors, for example, the redox-dependent modification of transcription factors as Activator Protein-1 (AP-1) [56]. AP-1 results from heretodimerization of c-Fos and c-Jun proteins, and this complex is important for proper induction of many genes involved in cellular damage protection and repair against ROS damage. Additionally, AP1 may be responsible for *Dnmt1* gene activation [57]. Moreover, cells that overexpress c-Fos showed increased Dnmt1expression and multiple drugs resistance phenotype, including drugs inducing oxidative stress [58]. In concordance with this, we observed that superoxide scavenger abrogated the increase in Dnmt1 mRNA and protein level (**Figure 1** **, D** and **E**) and global DNA hypermethylation (**Figure 1C**) in melanocytes submitted to anchorage restriction. Additionally, increased amounts of phosphorylated c-Jun was observed at nucleus in melan-a cells during adhesion impediment (data not showed), which is related with increased activity and DNA-binding capability [59]. In this way, alteration in AP-1 transcriptional factor phosphorylation and activity due to superoxide anion increase might change Dnmt1 expression and result in global DNA hypermethylation.

Proper attachment to extracellular matrix is essential to cell survival and loss of integrin engagement to extracellular matrix in non-transformed cells results in an apoptotic process namely *anoikis* [60]. *Anoikis* resistance is one of the acquired capabilities of tumorigenic cells to disseminate. Increased oncogene and decreased tumor suppressor genes expression can improve protection from *anoikis* [61], [62]. In this study, melan-a melanocyte lineage submitted to anchorage blockade presented Ras activation in the first 30 minutes (**Figure 2A**) and some authors have demonstrated that Ras signaling can affect gene expression through DNA methylation. Downregulation of tumor suppressor genes as E-cadherin and apoptosis-promoting protein Par-4, for example, was shown as result of their promoter hypermethylation induced by Ras [21], [63]. Moreover, it was shown that *K-Ras-*transformed NIH 3T3 fibroblasts have increased levels of Dnmt1 protein, which were associated with the *Fas* gene promoter hypermetylation [19]. Here we showed that FTI (farnesyltransferase inhibitor) impairs the increase in Dnmt1 protein level (**Figure 2B**) and global DNA hypermethylation (**Figure** **2C**) induced by anchorage restriction. Two of the major signaling pathways activated by Ras that are related to cancer and to changes in DNA methylation are PI3K-AKT and MEK-ERK [33], [34]. We rule out PI3K-AKT pathway involvement since its inhibition by Wortmannin and LY294002 had no effect on superoxide and Dnmt1 protein levels (**Figure S1**). In relation to MEK-ERK signaling pathway, we observed its activation during melan-a anchorage blockade (**Figure 4A**) and its inhibition impaired the increase both in superoxide (**Figure 5A and B**) and Dnmt1 protein level (**Figure 4B**) observed during adhesion impediment. In this way, Ras/Rac1/ERK pathway activation observed during loss of cell-matrix contacts may be involved in an epigenetic reprogramming caused by sustained oxidative stress condition. This process could then allow cells to adjust to new stressor environment, enabling them to survive. Supporting such hypotheses is the fact that several authors have shown increased superoxide levels after anchorage impediment [37], [38] and we demonstrated that its depletion during this process sensitized melanocyte and melanoma cells to *anoikis* [17], [37], [38]. Furthermore, cell lines obtained by sequential anchorage impediment cycles presented global and gene-specific epigenetic aberrant modifications [17]. We have also shown that anchorage impediment cycles performed in the presence of a superoxide scavenger gave rise to significant diminished number of clones compared to control after limiting dilution, suggesting that they are less *anoikis-*resistant. Moreover, these cell lines presented senescent-like morphology in culture differently of majority of control cell lines, which were obtained by the same process, but in absence of superoxide scavenger (**Figure 6** **, A** and **B**). One of senescence characteristics is that cells become larger, with flattened morphology and nucleus centrally located within cytoplasm. Besides that, senescent cells may be multinucleated and present positive β-Gal staining, common biomarker for senescence [64]. Although senescent-like cell lines obtained by adhesion impediment in the presence of superoxide scavenger present these features for long period, they can replicate in culture. Indeed there are some reports about cell lines that have senescent characteristics but replicate in culture [65]. Cellular senescence is a potent tumor suppressive mechanism that normally arrests cell proliferation and has been linked to aging and cellular response to stressful microenvironment conditions, including oxidative stress [66]. Some works show a central role of oxidative stress in senescence but they usually study the effects of hydrogen peroxide or non-selective antioxidants [67], [68]. In contrast, recent findings have showed that ROS activate signaling pathways and promote cell survival and longevity [69]. Here we observed specific superoxide effect on cellular senescent-like phenotype after the stressful condition that characterizes anchorage impediment since its depletion significantly delays senescent output. In this way, anchorage impediment cycles applied to melan-a melanocytes in an environment in which superoxide produced is depleted has another effect on cellular survival and behavior.

**Figure 7:**
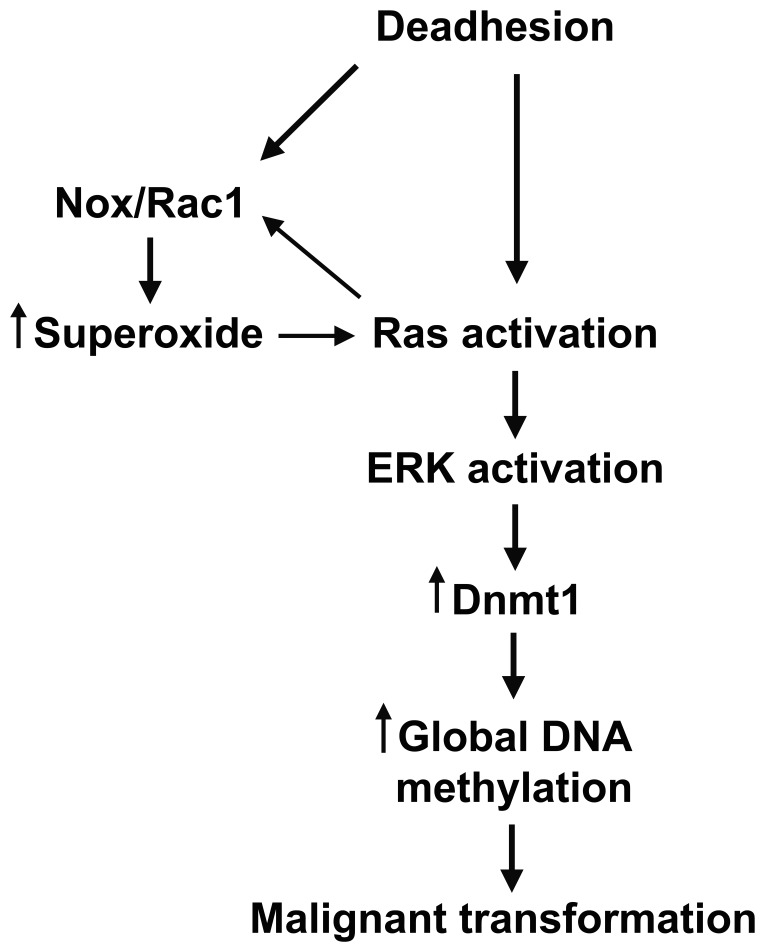
Stress condition and its relation with Ras/Rac1/MEK/ERK signaling pathway and epigenetic mechanisms regulation. Ras/Rac1/MEK/ERK signaling pathway seems to be regulated by and regulates superoxide anion production during melan-a anchorage blockade and its activation could be responsible for the high Dnmt1 protein level and changes in global DNA methylation during the loss of cell adhesion. Anchorage blockade might promote an epigenetic reprogramming in order to adapt the cells to the new microenvironmental condition and contribute to the acquisition of a malignant phenotype.

In our sustained stress model, Ras/Rac1/ERK signaling pathway regulates and is regulated by oxidative stress, specifically by superoxide anion. Besides that, this pathway is related to alterations in Dnmt1 protein levels and global DNA methylation (**Figure 7**). DNA methylation alterations caused by oxidative stress might change gene expression profile culminating in cellular phenotype and malignant transformation. It is possible that in human skin, conditions leading to increased production of ROS, such as UV radiation and inflammation, if in a sustained way, could result in epigenetic alterations, which may contribute to the melanocyte malignant transformation.

## Methods

### Ethics Statement

All animal work were conducted according to the recommendations in the Guide for the Care and Use of Laboratory Animals of the National Institutes of Health and were approved by Ethics Committee of Universidade Federal de São Paulo (CEP n° 0219-07).

### Cell Culture, Anchorage Blockade Assay and Cell Treatments

Melan-a is a non-tumorigenic cell line of pigmented melanocytes spontaneously derived from normal epidermal melanoblasts from embryos of inbred C57BL mice established by Bennett *et al* [70] and properly described in an article from *Int. J. Cancer* in 1987. The cells were cultured in RPMI 1640 pH 6.9 (Gibco, Carlsbad, CA) supplemented with 5% fetal bovine serum (v/v), antibiotics (Gibco) and 200 nM of phorbol myristate acetate (PMA, Sigma-Aldrich, Saint Louis, MO) at 37°C in humidified atmosphere of 5% CO_2_ and 95% air. For the anchorage blockade assay, melan- a cells (10^5^/ml) were cultured in suspension at 37°C for different times (10′, 30′ or 3 h) using 50 ml tubes or agarose-coated dishes.

Adherent cells (D0) or cells submitted to anchorage blockade for different times (D10’, D30 and D3 h) were treated or not with 50 µM of superoxide anion scavenger Mn(III)tetrakis(4-benzoic acid)porphyrin chloride (Mn(III)TBAP, Cayman, Ann Harbor, Michigan), with 12,5 µM of farnesyltransferase inhibitor (FTI, Bio Mol, Plymouth Meeting, PA), with 15 µM of ERK inhibitor U0126 (Calbiochem-Merck, Darmstad, Germany), with the PI3-kinase inhibitors LY294042 (5 µM) and wortmannin (0.5 µM) (Sigma) or with 100 µM of Rac1 inhibitor NSC23766 (Calbiochem-Merck, Darmstad, Germany in RPMI pH6.9 (Gibco) supplemented with 0.5% bovine serum, antibiotics (Gibco) and 200 nM of PMA (Sigma-Aldrich).

Non-tumorigenic melan-a cell line was also treated or not with 50 µM Mn(III)TBAP for 2 hours and subsequently, 1×10^4^ cells were submitted to sequential cycles of anchorage impediment in the presence or not of 50 µM Mn(III)TBAP as described [16].

### Measurement of Superoxide Anion (O_2_^.−^)

Adherent cells and cells submitted to anchorage blockade for different times were assayed for superoxide detection using dihydroethidium (DHE; Molecular Probes, Carlsbad, CA) and analyzed by flow cytometry (FACScalibur; Becton Dickinson, San Juan, CA and chemiluminescence was assessed immediately in a luminometer (Softmax Pro;Molecular Devices, Sunnyvale, CA, USA).

### 5-Methylcytosine Content Using 5-methylcytosine Specific Antibody

Global DNA methylation was analyzed by staining cells with a specific monoclonal antibody against 5-methylcytosine (Oncogene, La Jolla, CA) as previously described [5] and the data were analyzed by flow cytometry in a FACScan (Becton Dickinson).

### Western Blot

Whole-cell lysates were prepared using RIPA buffer (0.1% SDS (w/v), 0.5% NP-40 (v/v), 0.5% sodium deoxycholate (w/v), protease and phosphatase inhibitors) and used for Dnmt1 Western blot. Cytoplasmic protein extracts (150 mM of NaCl, 50 mM of Tris-HCl, 5 mM of EDTA, Igepal 1% (v/v) and protease and phosphatase inhibitors) were used for ERK Western blots. Membrane-enriched protein extracts (HEPES 25 mM, NaCl 150 mM, Igepal 1% (v/v), MgCl_2_ 10 mM_,_ EDTA 1 mM, protease and phosphatase inhibitors) were used for Rac1 Western blot. Protein lysates were resolved by SDS-PAGE and transferred onto PVDF membranes (Bio-Rad, Hercules, CA, USA). Specific antibodies were used followed by secondary antibody incubations, signals were visualized by chemoluminescence. The band intensities were measured using Processing and Analysis in Java, ImageJ 1.38b (Wayne Rasband, National Institute of Health, USA, http://rsb.info.nih.gov/ij/).

### Ras Activity Assay

Ras activation was determined using the Ras Activation Kit from Upstate (Upstate, Temecula, CA) as previously described [47].

### Tumorigenicity Assay

Cells from subconfluent monolayers were harvested after trypsin treatment, counted, and then suspended in PBS**.** The clones derived from sequential cycles of anchorage blockage in the presence or not of 50 µM Mn(III)TBAP (1×10^6^ cells) were subcutaneously injected in the flank of 6- to 8-week-old C57Bl/6 syngeneic female mice. Animals were kept under 12-hours daylight cycles, without food restriction, and checked daily for tumor development. The tumor volume (mm^3^) was measured using the formula: d^2^×D/2, where d represents the minimum diameter and D the maximum diameter. Each experimental group consisted of at least six animals.

### Cells Image Capture

The images were acquired by Olympus CK40/Olympus Singapore microscope using Image-pro 6.2/Media Cybernetics software®.

### Colony Formation Assay

Clones derived from sequential cycles of anchorage blockade in the presence or not of Mn(III)TBAP were subjected to clonogenic assay as previously described [71].

### DNA Sequencing

For DNA sequencing specific primers were designed for part of the exons 1 and 2 of H-Ras, K-Ras and N-Ras; and for whole Rac1 translated sequence (**Table S1**). The sequencing analysis was done using Genious® software.

### Real Time PCR

The relative expression level of *dnmt1* mRNA was determined by RT-qPCR in melan-a melanocytes adhered (D0) compared to those maintained in suspension for 3 hours (D3 h) treated or not with the superoxide scavenger Mn(III)TBAP (Mn). Dnmt1 oligonucleotides were 5′- GTGCCCTGCGTGGAGTCTGT-3′ (forward) and 5′- GTGGTTGTGCCGGTTCCCAGTG-3′ (reverse); for PCNA were 5′- TCGACACATACCGCTGCG-3′ (forward) and 5′- TAGAATTTTGGACATGCTGGTGA-3′ (reverse). To calculate the relative expression, melan-a was used as the calibrator using the formula RQ = 2 -DDCt, where DDCt = DCtsample − DCtmelan-a, DCt = Ctdnmt1 − CtPCNA.

### Statistical Analysis

Non-paired non-parametric Student’s *t*-test was used to analyze differences between the means using GraphPad Prism version 4 for Windows (GraphPad, San Diego, CA). The significance level was established at p<0.05.

## Supporting Information

Figure S1 **Increased level of Dnmt1 during melanocyte deadhesion does not involve PI3K-Akt signaling pathway.** Superoxide level was determined by luminescence assay using coelenterazine in melan-a melanocytes adhered (D0) or maintained in suspension for 3 h (D3 h), treated or not (NT) with 0.5 µM Wortmannin (Wn) (**A**) or by flow cytometry using DHE in melan-a melanocytes adhered (D0) or maintained in suspension for 3 h (D3 h), treated or not (NT) with 5 µM LY294042 (**B**). Dnmt1 protein expression evaluated by Western blot in the same cells treated or not with 0.5 µM Wortmannin (Wn) (**C**) or not with 5 µM LY294042 (**D**). ****p<0.0001.
(TIF)

Table S1 **Primers used for sequencing.**
(DOCX)
