# Correction: Ras and Rac1, Frequently Mutated in Melanomas, Are Activated by Superoxide Anion, Modulate Dnmt1 Level and Are Causally Related to Melanocyte Malignant Transformation

**DOI:** 10.1371/journal.pone.0124983

**Published:** 2015-04-13

**Authors:** Fernanda Molognoni, Fabiana Henriques Machado de Melo, Camila Tainah da Silva, Miriam Galvonas Jasiulionis

The image for the Mn2 panel of Fig 6B is incorrect. Please see the complete, corrected Fig 6 here.

**Fig 6:**
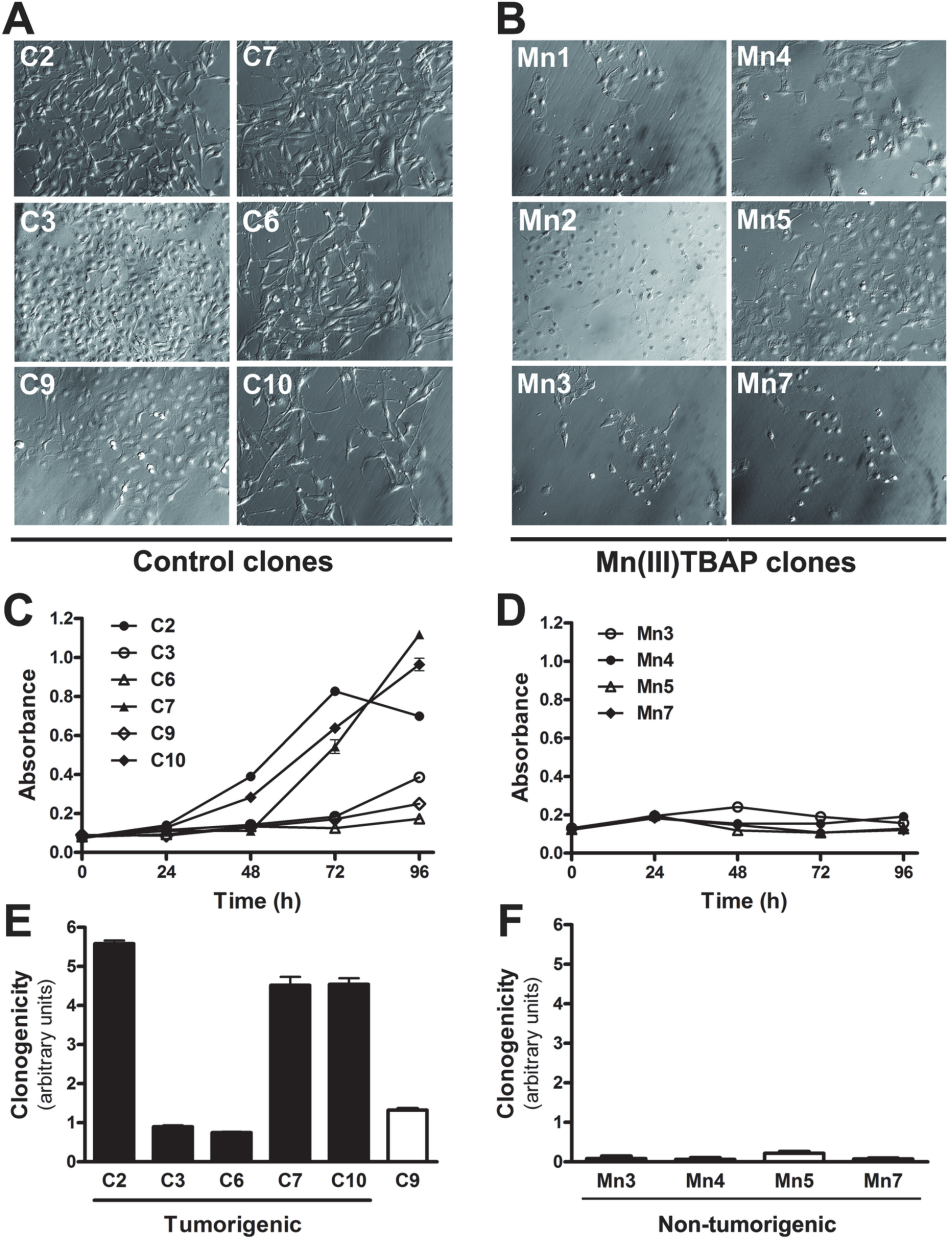
Abrogating superoxide anion during sequential cycles of anchorage restriction results in clones with reduced cell proliferation and clonogenicity and increases the time required to malignant conversion. Melan-a melanocytes were submitted to sequential cycles of anchorage impediment in the presence (Mn clones) or not (control clones) of 50 μM Mn(III)TBAP. Almost control clones showed spindle morphology (**A**) whereas Mn clones senescent-like aspect (**B**). Cell proliferation was analyzed by MTT assay in control (**C**) and Mn clones (**D**). The clonogenic capability of control (**E**) and Mn clones (**F**) was evaluated before escape from senescent-like phenotype by plating 200 cells on 60 mm-dishes. After 9 days, the cell number was estimated by measuring the absorbance after lysing the cells stained with Toluidine blue.
