# Supplementary material for: Ras and Rac1, Frequently Mutated in Melanomas, Are Activated by Superoxide Anion, Modulate Dnmt1 Level and Are Causally Related to Melanocyte Malignant Transformation
Source: PLoS One. 2013 Dec 16;8(12):e81937. doi: 10.1371/journal.pone.0081937 (PMC3864863; doi:10.1371/journal.pone.0081937)
Supplement: Table S1 — Primers used for sequencing. (DOCX) [file pone.0081937.s002.docx]

**Table S1**

| **Primers for sequencing** | **Forward** | **Reverse** |
| --- | --- | --- |
| **Rac1** (translated region) | 5’GATGCAGGCCATCAAGTGTGTGGT3’ | 5’TCCCTAAGATCAAGCTTCGTCCCCC3’ |
|  | 5’GTGGGGACGAAGCTTGATCTTAGGG3’ | 5’ACAGGTGCGTTACATGGCAACGTG3’ |
|  | 5’CACGTTGCCATGTAACGCACCTGT3’ | 5’ACAGGACGCACTTCTACTGAGCTAC3’ |
| **H-Ras** (exons 2, 3, and 4) | 5’CAGCCGCTGTAGAAGCTATGACAG3’ | 5’AGCAGCCAGGTCACACTTGTTG3’ |
| **K –Ras** (exons 2, 3, and 4) | 5’GAGAGAGGCCTGCTGAAAATGAC3’ | 5’ACCAGGACCATAGGCACATCTTC3’ |
| **N-Ras** (exons 2, 3, and 4) | 5’CTCCAACAGCTTCTCAGGTTGAAGT3’ | 5’GTCCTTGTTGGCAAGTCACACT3’ |

**Table S1. Primers used for sequencing**
